# Mechanisms Involved in Therapeutic Effects of *Scutellaria baicalensis* Georgi in Oral Squamous Cell Carcinoma Based on Systems Biology and Structural Bioinformatics Approaches

**DOI:** 10.1155/2024/1236910

**Published:** 2024-01-30

**Authors:** Zeynab Bayat, Tina Mazaheri, Homa Farhadifard, Amir Taherkhani

**Affiliations:** ^1^Department of Oral and Maxillofacial Medicine, School of Dentistry, Hamadan University of Medical Sciences, Hamadan, Iran; ^2^Department of Orthodontics, School of Dentistry, Hamadan University of Medical Sciences, Hamadan, Iran; ^3^Research Center for Molecular Medicine, Hamadan University of Medical Sciences, Hamadan, Iran

## Abstract

**Objective:**

Oral squamous cell carcinoma (OSCC) is the most frequent oral cancer, constituting more than 90% of all oral carcinomas. The 5-year survival rate of OSCC patients is not satisfactory, and therefore, there is an urgent need for new practical therapeutic approaches besides the current therapies to overcome OSCC. *Scutellaria baicalensis* Georgi (SBG) is a plant of the family Lamiaceae with several pharmaceutical properties such as antioxidant, anti-inflammatory, and anticancer effects. Previous studies have demonstrated the curative effects of SBG in OSCC.

**Methods:**

A systems biology approach was conducted to identify differentially expressed miRNAs (DEMs) in OSCC patients with a dismal prognosis compared to OSCC patients with a favorable prognosis. A protein interaction map (PIM) was built based on DEMs targets, and the hub genes within the PIM were indicated. Subsequently, the prognostic role of the hubs was studied using Kaplan-Meier curves. Next, the binding affinity of SBG's main components, including baicalein, wogonin, oroxylin-A, salvigenin, and norwogonin, to the prognostic markers in OSCC was evaluated using molecular docking analysis.

**Results:**

Survival analysis showed that overexpression of CAV1, SERPINE1, ACTB, SMAD3, HMGA2, MYC, EIF2S1, HSPA4, HSPA5, and IL6 was significantly related to a poor prognosis in OSCC. Besides, molecular docking analysis demonstrated the Δ*G*_binding_ and inhibition constant values between SBG's main components and SERPINE1, ACTB, HMGA2, EIF2S1, HSPA4, and HSPA5 were as <-8.00 kcal/mol and nanomolar concentration, respectively. The most salient binding affinity was observed between wogonin and SERPINE1 with a criterion of Δ*G*_binding_ < −10.02 kcal/mol.

**Conclusion:**

The present results unraveled potential mechanisms involved in therapeutic effects of SBG in OSCC based on systems biology and structural bioinformatics analyses.

## 1. Introduction

Oral carcinoma is the most frequent cancer in the head and neck area [1]. Oral squamous cell carcinoma (OSCC) represents 90000 of all types of oral cancers [2], ranking sixth among all forms of carcinomas worldwide [3]. Tobacco and alcohol consumption, as well as human papillomavirus (HPV), are the most common independent risk factors for OSCC [4]. Chemotherapy, surgery, radiotherapy, and immunotherapy are commonly used to treat OSCC [5]. However, the 5-year survival rate of patients with OSCC has remained at 50 to 60% in the primary stages, and it decreases to 30 to 40% of patients in the late stages of the disease [6]. Thus, there is an urgent need for novel therapies in combination with the current therapeutic approaches to overcome OSCC. Likewise, targeted therapies use specific drugs per the tumor location, leading to high selectivity, low toxicity, and high therapeutic outcomes in cancer treatment [7, 8].

Medicinal plants have long been used to flavor foods and to treat/prevent several human disorders, and herbal nutraceuticals are in the interest of many medicians for primary healthcare. It is worth mentioning that most of the approved drugs are organic-based agents [9]. *Scutellaria baicalensis* Georgi (SBG) is a plant of the family Lamiaceae with many useful pharmaceutical features such as anticancer, antimicrobial, antioxidant, and anti-inflammatory properties [10, 11]. Its dried root, Huang Qin, is one of the primary medicinal sources in traditional Chinese medicine [12, 13]. SBG has also been confirmed by the European Pharmacopoeia (EP 9.0) and British Pharmacopoeia (BP 2018) [14]. Huang Qin has shown curative effects in allergic disorders, respiratory and gastrointestinal diseases, hepatitis, colitis, and pneumonia [15, 16]. In addition, several studies have indicated that SBG might serve as a vital herbal source of therapeutic components for treating OSCC due to its outstanding properties and low side effects [17–19]. Secondary metabolites in plants are active agents responsible for the biological activities of herbs [20]. According to the study by Hou et al. [21], five flavonoids, including baicalein, wogonin, oroxylin-A, salvigenin, and norwogonin, are closely associated with the therapeutic effects of SBG against OSCC. Besides, these components have shown inhibitory effects against cancer cell development [22].

Herein, it was hypothesized that baicalein, wogonin, oroxylin-A, salvigenin, and norwogonin might target essential genes mediating poor prognosis in patients with OSCC. Therefore, an integrated bioinformatics study was executed to identify negative prognostic markers in OSCC patients. The prognostic markers were assigned as potential targets for SBG components. Subsequently, the binding affinity of baicalein, wogonin, oroxylin-A, salvigenin, and norwogonin to the binding sites of targets was evaluated using molecular docking analysis. Therefore, the present study followed two parts: (1) a systems biology study for identifying potential biomarkers associated with a dismal prognosis in patients with OSCC and (2) structural bioinformatics analysis to indicate binding affinities between prognostic markers and SBG active components.

The systems biology section of the study was carried out by reanalyzing the high-throughput sequencing GSE52633 dataset developed by Yoon et al. [23] to identify differentially expressed miRNAs (DEMs) in primary OSCC tissue samples achieved from patients with poor 5-year survival compared to early-OSCC tissue specimens obtained from patients with favorable 5-year survival. After that, validated targets of DEMs were indicated, a protein interaction map (PIM) was constructed, and hub genes within the PIM were identified. Next, the prognostic role of the hubs was evaluated using the Kaplan-Meier curves.

## 2. Materials and Methods

### 2.1. MicroRNA Dataset Recovery and Statistical Analysis

The high-throughput sequencing miRNA dataset GSE52633 [23] was downloaded as a TXT file from the NCBI GEO, available from http://www.ncbi.nlm.nih.gov/geo [24]. The dataset included early-OSCC tissue samples collected from patients with poor 5-year survival (*n* = 10) and favorable 5-year survival (*n* = 10) based on the GPL16791 platform (Illumina HiSeq 2500 (Homo sapiens)).

The Min–Max method was used for normalizing the dataset using the R programming version 4.2.2 [25]. An advanced multivariate statistical analysis, orthogonal partial least squares discriminant analysis (*OPLS*-*DA*) [26], was utilized to indicate DEMs between the studied groups; this was done using the “genefilter,” “limma,” and “ropls” packages from the R language. miRNAs with a *p* value < 0.05 and fold change (FC) difference of >2 or <1/2 were assigned significant features between the studied groups. Further, the Shiny server, available from https://huygens.science.uva.nl/ [27], illustrated the volcano plot of the dataset GSE52633.

### 2.2. Networking and Gene Set Enrichment Analysis

Validated targets of DEMs were identified using the mirTarBase database [28]. Only the genes that were experimentally validated using at least one of the robust evidence methods (including reporter assay, western blot, and qPCR) or at least two of the less intense evidence approaches (including microarray, NGS, pSILAC, and CLIP-Seq) were assigned targets of DEMs. Possible interactions among DEMs targets were indicated using the STRING version 11.5 knowledge base, available from http://string-db.org [29]. The STRING provides valuable information about billions of interactions between millions of proteins achieved from thousands of organisms. The Cytoscape 3.9.1, available from https://cytoscape.org/ [30], visualized the PIM and calculated the centrality of the nodes within the graph. Unconnected proteins were eliminated from the network [31]. Subsequently, the genes with degree and betweenness centralities above the average of the nodes in the network were considered hub genes. They were evaluated for their possible role in the prognosis of patients with OSCC. Modules within the PIM were demonstrated using the MCODE plugin [32] to see whether the prognostic markers are involved in clusters associated with pathways and biological processes (BPs) mediating the pathogenesis of OSCC patients with poor prognoses. Condensed regions with the following features were considered significant modules and were selected for further pathway and BP analysis: (1) MCODE score > 3, (2) the number of genes > 10 [33], and (3) including prognostic marker(s). Significant pathways and BPs affected by the clusters were explored using the g:Profiler tool, available from https://biit.cs.ut.ee/gprofiler/gost [34]. A cutoff condition was set to false discovery rate (FDR) < 0.05 and the number of enriched genes within the term > 10.

### 2.3. Survival and Boxplot Analyses

Kaplan-Meier curves were achieved using the GEPIA2 database, available from http://gepia2.cancer-pku.cn/#survival [35], to evaluate the prognostic impact of the hub genes in OSCC. The prognostic role of the genes with the log-rank test and hazard ratio (HR) *p* value < 0.05 were considered significant. The GEPIA2 applies powerful analyses on RNA sequencing data from The Cancer Genome Atlas [36] and Genotype-Tissue Expression [37] databases, leading to reliable results for survival and boxplot analyses in patients with cancer as compared to healthy individuals. In addition, the expression patterns of negative markers in OSCC tissues and healthy control samples were evaluated using relevant data from the GEPIA2 database.

### 2.4. Structural Preparation of the Ligands and Receptors

The hub genes with a significant role in the prognosis of patients with OSCC were assigned possible targets for baicalein, wogonin, oroxylin-A, salvigenin, and norwogonin. Most of the targets' three-dimensional (3D) structures were achieved from the RCSB database, available from https://www.rcsb.org [38]. For receptors with no structural data in the RCSB, the similarity of their templates was checked in the PDB. For targets with templates with a similarity above 30% [39], homology modeling was performed using the SWISS-MODEL web server, available from https://swissmodel.expasy.org/ [40]. Otherwise, threading modeling was executed using the I-TASSER server, available from https://zhanggroup.org/I-TASSER/ [41].

Subsequent to the initial modeling, a refinement process was applied to enhance the structure of the modeled proteins, utilizing the GalaxyWEB server, accessible at https://galaxy.seoklab.org/index.html [42]. Subsequently, the integrity of the modeled proteins' structures underwent additional scrutiny to ascertain the dependability of the outcomes. To this end, assessments were conducted using the UCLA-DOE LAB – SAVES v6.0 web server encompassing ERRAT [43], Verify 3D [44], and PROCHECK [45] analyses, accessible at https://saves.mbi.ucla.edu/. Additionally, the protein structure analysis (ProSA) tool [46] was employed to provide an overarching evaluation of the overall quality of the predicted structures.

The energy minimization (EM) process was applied on all proteins before molecular docking analysis using the Swiss-PdbViewer version 4.1.0, available from https://spdbv.unil.ch [47]. The structures of ligands were obtained as SDF files and converted into PDF formats, followed by EM [48, 49]. Kollman charge and polar hydrogens were added to the structures of receptors. Besides, local charge and rotational motion were included in ligands. Finally, the PDBQT files were built for the proteins and small molecules using the MGL tools [50].

### 2.5. Molecular Dockings, Dynamics, and Interaction Mode Analyses

A Windows-based PC with the following features was used for *in silico* analyses: system type, 64-bit; installed RAM, 64 GB DDR5; and processor, Intel 24-Core i9-13900KF. The Gibbs free energy of binding (Δ*G*_binding_) between ligands and receptors was calculated using the AutoDock 4.0 software. A total of 100 independent runs were set for each component. The most negative Δ*G*_binding_ value in the root mean square deviation (RMSD) table was considered binding energy between ligands and receptors [50].

Discovery Studio Client (DSC) version 16.1.0.15350 was used to uncover interactions between SBG active compounds and OSCC prognostic markers. Molecular dynamics (MD) was executed in a 100-nanosecond (ns) computer simulation using the DSC tool to evaluate the structural stability of the most salient complex in comparison to the reference drug [51–53]. In configuring the computer simulations, the specified parameters were as follows: orthorhombic cell shape, 10 Å minimum distance from the boundary, water as the solvent, 310 K target temperature, CHARMM as the force field, the explicit periodic boundary for solvation model, and a point charge distribution [54].

Notably, the preeminent molecular docking outcome, elucidating the interaction between receptors and ligands, was meticulously juxtaposed with that of a reference pharmaceutical agent. Furthermore, the results derived from MD simulations for the most prominent complex were systematically contrasted with those of the unbound receptor and the receptor inhibited by the reference drug.

## 3. Results

### 3.1. DEMs and Their Targets in OSCC Patients with Poor Prognosis

**Figure 1:**
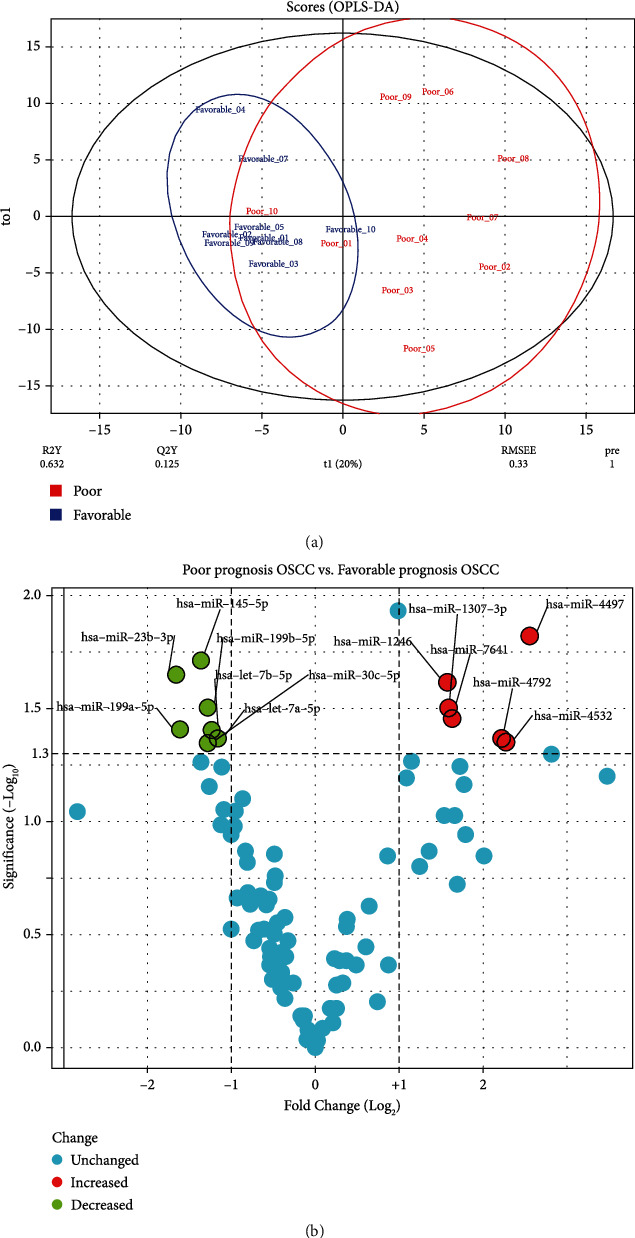
(a) The score plot in the predictive (*X*-axis) and orthogonal (*Y*-axis) components of miRNA dataset GSE52633 achieved from the OPLS-DA. (b) The volcano plot of miRNAs in the primary OSCC tissues compared with the normal samples. OPLS-DA: orthogonal partial least squares discriminant analysis; OSCC: oral squamous cell carcinoma.

The OPLS-DA model significantly differentiated early-OSCC tissue samples with poor 5-year survival from that with favorable 5-year survival (*R*^2^*X* = 0.536, *R*^2^*Y* = 0.632, and *Q*^2^ = 0.125) Figure 1(a). Thirteen DEMs with the criteria of *p* value < 0.05 and |Log2 FC| > 1, including six upregulated and seven downregulated DEMs, were identified between the studied groups (Figure 1(b) and Table 1). A total of 476 genes were indicated as experimentally validated targets of DEMs.

### 3.2. Topological and Functional Analyses of Protein-Protein Interaction Network

**Figure 2:**
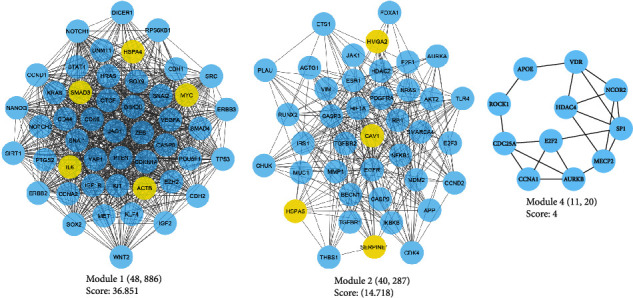
The MCODE plugin identified three clusters within the PPI network associated with early-OSCC patients with poor prognoses. PPI: protein-protein interaction; OSCC: oral squamous cell carcinoma.

**Figure 3:**
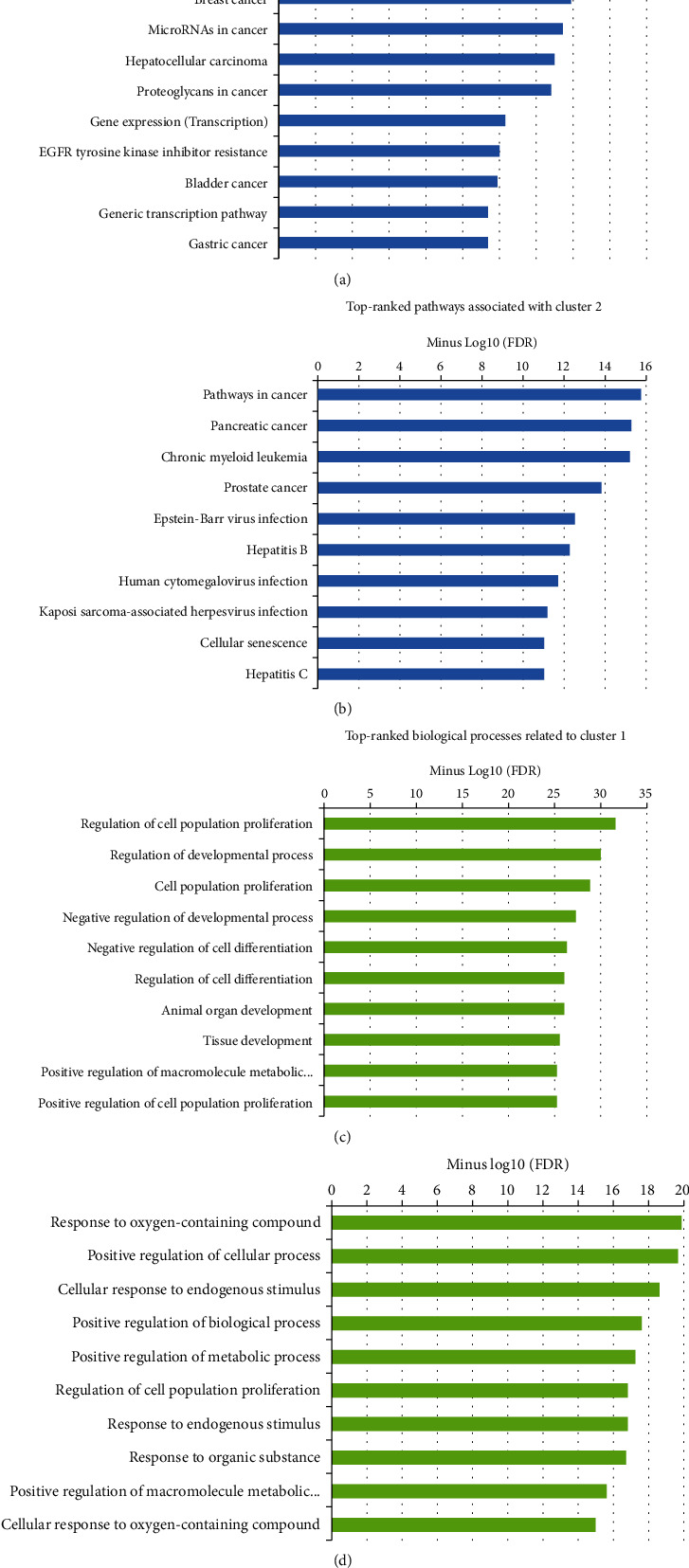
The most significant pathways associated with (a) cluster no. 1 and (b) cluster no. 2 and biological processes related to (c) cluster no. 1 and (d) cluster no. 2 regulated by the SBG components in patients with oral squamous cell carcinoma. The *X*-axis shows the minus value of the Log10 FDR. *Y*-axis demonstrates the name of the term. FDR: false discovery rate; SBG: Scutellaria baicalensis Georgi.

The interactions between DEM targets were illustrated with a confidence score of ≥0.4 using the STRING database. Disconnected nodes were removed from the graph, and the Cytoscape demonstrated the protein-protein interaction (PPI) network, including 442 vertexes and 5226 edges. Topological analysis revealed 88 proteins with the degree and betweenness centralities above the average value of the nodes and, therefore, assigned hub proteins in the PPI network associated with the pathogenesis of OSCC patients with poor prognoses (Supplementary Table 1).  Table 2 provides the first 30 genes according to the degree of the nodes. The average values for betweenness and degree were recorded as 0.0038 and 23.65, respectively. Three modules were identified in the PPI network with a number of genes > 10 and MCODE score above three (cluster nos. 1, 2, and 4). By performing survival analysis, it was found that module 1 and module 2 contain prognostic markers associated with a poor prognosis in OSCC patients (Figure 2). A total of 427 BPs and 47 pathways were enriched by cluster 1. Besides, cluster 2 was involved in 265 BPs and 35 pathways. Top-10 significant pathways and BPs affected by clusters 1 and 2 are presented in Figure 3.

### 3.3. Survival and Expression Analyses

**Figure 4:**
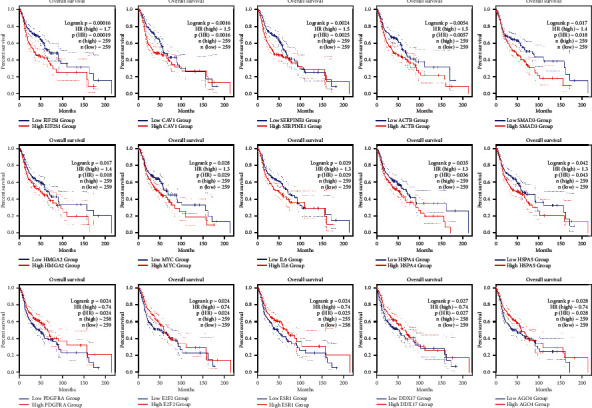
Prognostic role of EIF2S1, CAV1, SERPINE1, ACTB, SMAD3, HMGA2, MYC, IL6, HSPA4, HSPA5, PDGFRA, E2F2, ESR1, DDX17, and AGO4 was significant in patients with OSCC. The *X*-axis and *Y*-axis represent the survival time of OSCC patients and the survival probability, respectively. The dotted lines are 95% confidence intervals. OSCC: oral squamous cell carcinoma.

**Figure 5:**
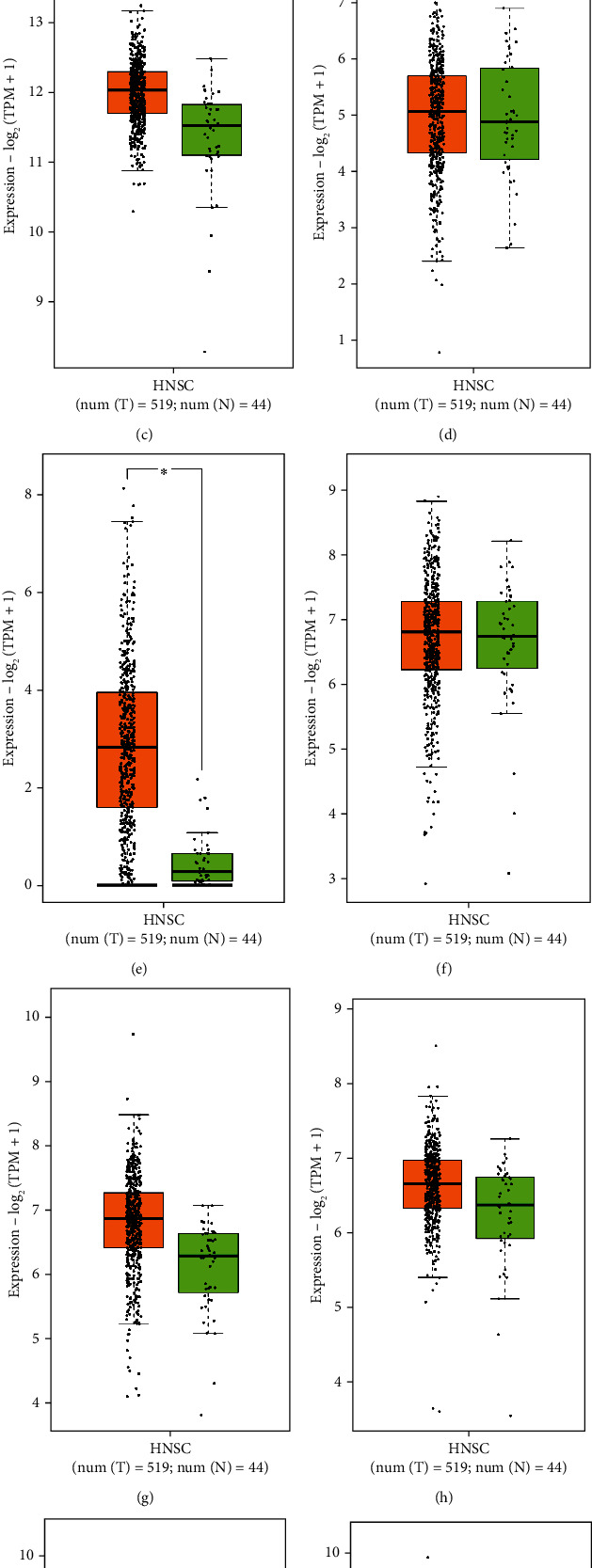
Gene expression pattern of negative markers in OSCC from the boxplot analysis achieved from the GEPIA2 database based on 519 OSCC samples (orange color) and 44 normal tissues (green color). The data show overexpression of *CAV1* (a), *SERPINE1* (b), *ACTB* (c), *SMAD3* (d), *HMGA2* (e), *MYC* (f), *EIF2S1* (g), *HSPA4* (h), and *HSPA5* (i) and downregulation of IL6 (j) in OSCC. However, SMAD3 and MYC demonstrate a mild overexpression in OSCC. OSCC: oral squamous cell carcinoma.

The Kaplan-Meier curves revealed that the upregulation of ten genes, including CAV1, SERPINE1, ACTB, SMAD3, HMGA2, MYC, EIF2S1, HSPA4, HSPA5, and IL6, was significantly related to a dismal outcome in patients with OSCC. Besides, the overexpression of PDGFRA, E2F2, ESR1, DDX17, and AGO4 was associated with a favorable prognosis in OSCC patients (log-rank test and HR *p* values < 0.05) (Figure 4 and Table 3). Moreover, the boxplot analysis revealed that all negative markers in OSCC, except IL6, exhibited higher expression in OSCC tissues compared to the normal samples (Figure 5).

### 3.4. Structural Preparation and Binding Site Detection

The 3D coordinates of SERPINE1, ACTB, SMAD3, MYC, EIF2S1, HSPA5, and IL6 were available from the RCSB database. SWISS-MODEL web server was used to model the structure of HSPA4. Further, the structures of CAV1 and HMGA2 were prepared using the I-TASSER tool.

Following the implementation of the model refinement process and a comprehensive evaluation of the modeled proteins' structures, HMGA2 successfully cleared all assessment criteria. Conversely, HSPA4 did not meet the requirements of the Verify 3D analysis. Nevertheless, the overall structural integrity of HSPA4 was verified through the ProSA web server, justifying its inclusion in the study for subsequent molecular docking analysis. On the other hand, CAV1 did not meet the specified quality assessment parameters, leading to its exclusion from further analysis. For a detailed overview of all model assessment analyses, refer to Supplementary File 1.

Different strategies were used to indicate the central residues involved in the binding sites of the receptors. The DSV tool revealed the interacting residues of SERPINE1 with the ligand inside the PDB file of the protein (PDB entry, 1A7C). The HMGA2 binding residues were indicated using the UniProt database. Besides, the CASTp server unraveled interacting residues of EIF2S1.  Table 4 presents the (1) different sources used for achieving 3D structures of receptors, (2) strategy for identifying binding sites, (3) main residues in binding sites, and (4) grid box settings. The Gasteiger charge assigned to baicalein exhibited a value of −0.0002, whereas for wogonin, oroxylin-A, salvigenin, and norwogonin, the Gasteiger charges were each registered at −0.0001. Comprehensive details of the Kollman charges applied to the proteins are elaborated upon in Table 5 [51, 55].

### 3.5. Binding Affinity Assessment

The higher binding affinity between ligands and receptors results in a smaller Δ*G*_binding_ value. It has been demonstrated that Δ*G*_binding_ < −7.00 kcal/mol shows a robust binding affinity [67]. The results show the Δ*G*_binding_ and inhibition constant (*K*_*i*_) values between SBG components and SERPINE1, ACTB, HMGA2, EIF2S1, HSPA4, and HSPA5 were calculated as <−8.00 kcal/mol and nanomolar scale, respectively. Therefore, these receptors were assigned as potential targets of SBG components. Thus, inhibiting these proteins might be involved in the therapeutic effects of SBG in patients with OSCC. All Δ*G*_binding_ and *K*_*i*_ values between SBG components and receptors are presented in Tables 6 and 7, respectively.

The most salient binding affinity was observed between wogonin and SERPINE1 with the criteria of Δ*G*_binding_ and *K*_*i*_ values as −10.02 kcal/mol and 45.08 nM, respectively. Following the selection criteria, colforsin (PubChem ID, 47936; DrugBank ID, DB02587) was indicated as a positive control inhibitor for SERPINE1, leveraging information from the DrugBank database accessible at https://go.drugbank.com/ [68]. The calculated Δ*G*_binding_ score and *K*_*i*_ value, representing the binding affinity between SERPINE1 and colforsin, stood at 11.3 kcal/mol and 5.17 nM, respectively.

### 3.6. Interaction Mode Analysis and MD Simulation

**Figure 6:**
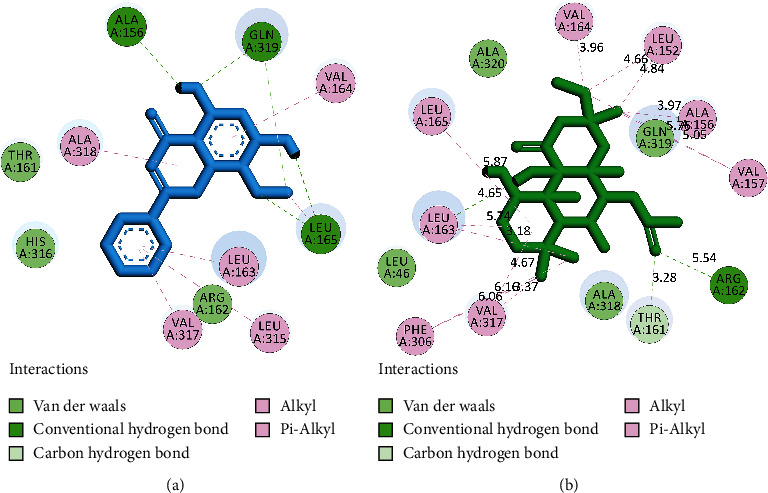
Two-dimensional view of (a) wogonin and (b) colforsin inside the SERPINE1 active site. SERPINE1: plasminogen activator inhibitor 1.

Interactions between wogonin and SERPINE1 were demonstrated utilizing the DSC tool. Accordingly, wogonin formed five hydrogen bonds and six hydrophobic interactions with the residues of SERPINE1 (Figure 6(a)). In comparison, colforsin exhibited two H-bonds and eight hydrophobic interactions with residues incorporated inside the SERINE1 active site (Figure 6(b)).

**Figure 7:**
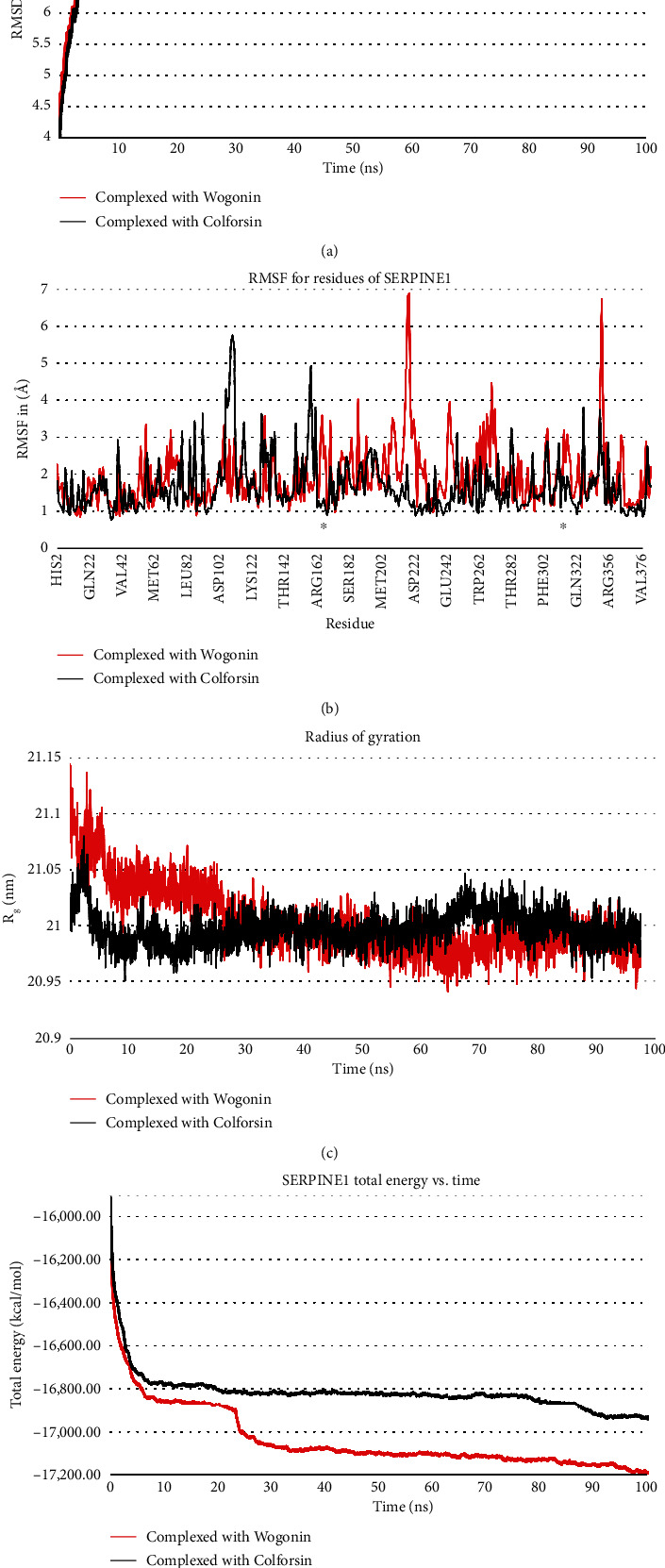
The influence of wogonin and colforsin on SERPINE1 backbone atoms was investigated through a meticulous 100 ns MD simulation, with a specific emphasis on (a) RMSD, (b) RMSF, (c) ROG, and (d) total energy plots. Notably, asterisks within the RMSF plots signify the locations of the active site on the receptor. SERPINE1: serpin family E member 1; RMSF: root mean square fluctuation; RMSD: root mean square deviations.

Moreover, the MD simulations delved into the behavior of SERPINE1 when complexed with wogonin and colforsin. The root mean square deviation (RMSD) plot (Figure 7(a)) revealed a notably more stable structure of SERPINE1 when in complex with colforsin compared to wogonin. Specifically, the SERPINE1 backbone atoms achieved stability after approximately 10 ns of computer simulation, maintaining at around 7 Å in the presence of colforsin. In contrast, when complexed with wogonin, stability was attained after 30 ns, with the protein's backbone atoms stabilizing at approximately 8 Å. Analyzing the root mean square fluctuation (RMSF) plot (Figure 7(b)), it became apparent that the backbone atoms of SERPINE1 within the enzyme's active site exhibited greater stability when complexed with colforsin in comparison to wogonin. Further MD analyses unveiled that during the initial 30 ns of the simulation, the radius of gyration (ROG) of SERPINE1 was lower in the presence of colforsin than wogonin. Between the simulation times of 30-60 ns, the ROGs of SERPINE1 were approximately equivalent when complexed with both ligands. Additionally, within the simulation timeframe of 60-90 ns, the ROG for SERPINE1 was lower when bound to wogonin compared to colforsin (Figure 7(c)). Interestingly, the total energy of SERPINE1 remained consistently lower throughout the entire simulation period when engaged with wogonin compared to the reference drug (Figure 7(d)).

## 4. Discussion

Accumulating evidence suggests that SBG is a valuable plant source with curative effects in OSCC [17–19]. The present study performed an integrated bioinformatics analysis to identify potential mechanisms involved in the therapeutic effects of SBG in OSCC. Our systems biology analysis indicated that CAV1, SERPINE1, ACTB, SMAD3, HMGA2, MYC, EIF2S1, HSPA4, HSPA5, and IL6 upregulation is significantly associated with a poor prognosis in patients with OSCC. Additionally, structural bioinformatics analysis showed that SBG active metabolites had a considerable binding affinity to SERPINE1, ACTB, HMGA2, EIF2S1, HSPA4, and HSPA5 (Δ*G*_binding_ < −8 kcal/mol and *K*_*i*_ value at nanomolar concentration). The most salient binding affinity was observed between wogonin and SERPINE1 with the criteria of Δ*G*_binding_ < −10.02 kcal/mol and *K*_*i*_ value as 45.08 nM. Wogonin exhibited five hydrogen and six hydrophobic interactions with Ala156, Leu163, Val164, Leu165, Leu315, Val317, Ala318, and Gln319 within the SERPINE1 binding site.

Previous reports have indicated anti-inflammatory, antioxidant, immunomodulatory, and antitumor properties for wogonin [69]. Wogonin also has a chemosensitizer effect in cancer chemotherapy. It has induced cancer cell apoptosis when combined with cisplatin, doxorubicin, etoposide, and 5-FU [70, 71]. Wogonin conducts its antitumor activities through several molecular mechanisms [72, 73]. You et al. [74] reported that wogonin downregulated the epithelial-mesenchymal transition (EMT) in colorectal cancer cells. Wogonin also diminished the transcriptional coactivator YAP1 and interferon regulatory factor 3 (IRF3) expression in vitro and in vivo, leading to the Hippo signaling pathway upregulation. Zhang et al. [75] demonstrated that wogonin elevated the apoptosis process in pancreatic cancer cells by downregulating the Akt signaling pathway, leading to increased gemcitabine sensitivity to pancreatic cancer cells. Flow cytometry and western blotting methods approved the study's results by Zhang et al. [75]. Tsai et al. [76] reported that wogonin increased reactive oxygen species (ROS) generation and ER stress in human glioma cells, resulting in caspase-9 and caspase-3 hyperactivity and cancer cell apoptosis. In addition, Zhao et al. [77] demonstrated wogonin's antiproliferative and apoptotic activities in ovary cancer cells.

Herein, other mechanisms were identified to be involved in the therapeutic effects of wogonin in OSCC. It was found that wogonin can potentially inhibit five genes associated with a poor prognosis in patients with OSCC. Wogonin demonstrated salient binding affinities to SERPINE1 (Δ*G*_binding_ < −10.02 kcal/mol), ACTB (Δ*G*_binding_ < −9.22 kcal/mol), HMGA2 (Δ*G*_binding_ < −8.8 kcal/mol), EIF2S1 (Δ*G*_binding_ < −8.91 kcal/mol), and HSPA4 (Δ*G*_binding_ < −9.09 kcal/mol).

SERPINE1 (serpin family E member 1) is a serine proteinase inhibitor involved in tissue plasminogen activator (tPA) and urokinase (uPA) inhibition [78, 79]. The oncogenic role and overexpression of SERPINE1 have been demonstrated in multiple cancers [80]. In this regard, it has been reported that SERPINE1 is upregulated in gastric cancer and mediates cancer cells' proliferation and invasion behavior [81]. SERPINE1 is also highly expressed in breast cancer, leading to the metastasis of tumor cells [82]. Likewise, accumulating evidence has confirmed the SERPINE1 overexpression in OSCC [82–84]. Zhao et al. [85] demonstrated that SERPINE1 is a proproliferative oncogenic factor in OSCC cells and is negatively regulated by miR-167. Zhao et al. [85] concluded that targeting SERPINE1 by miR-167 diminished cellular viability and proliferation, leading to apoptosis in OSCC. Therefore, it might be suggested that similar mechanisms are involved in the therapeutic effects of wogonin and miR-167 in OSCC, although this requires confirmation.

Our network analysis revealed that cluster no. 1 and cluster no. 2 include nine genes mediating a dismal prognosis in patients with OSCC. Therefore, targeting prognostic genes in these clusters might be suggested to regulate pathways and BPs involved in the etiology of OSCC patients with poor prognoses. GO annotation analysis demonstrated that the “regulation of cell population proliferation” (GO:0042127) is significantly affected by cluster no. 1 and cluster no. 2, consistent with the findings of Zhao et al. [85].

## 5. Conclusion

Collectively, a total of 13 DEMs (upregulated = six; downregulated = seven) were identified in early-OSCC patients with a poor prognosis compared to early OSCC with a favorable prognosis. A PPI network was constructed based on DEMs targets, including 442 genes and 5226 edges. Kaplan-Meier curves demonstrated that overexpression of ten hub genes, including CAV1, SERPINE1, ACTB, SMAD3, HMGA2, MYC, EIF2S1, HSPA4, HSPA5, and IL6, was significantly associated with a dismal prognosis in OSCC. It is suggested that the SBG's main components, including baicalein, wogonin, oroxylin-A, salvigenin, and norwogonin, have high binding affinities to prognostic markers in OSCC. A remarkable binding affinity was computed between wogonin and SERPINE1, meeting the criterion of Δ*G*_binding_ < −10.02 kcal/mol, indicative of a significant and stable interaction. SERPINE1 suppression has diminished OSCC proliferation, and therefore, it might be suggested that downregulating cell proliferation is one of the mechanisms mediating the curative effects of wogonin in OSCC. The present results uncovered prognostic markers and molecular mechanisms mediating poor prognoses in patients with OSCC. Likewise, targeting prognostic markers could be a potential mechanism of SBG, resulting in curative aspects in patients with OSCC.

## Figures and Tables

**Table 1:** Thirteen miRNAs were indicated to be differentially expressed in early-OSCC patients with poor survival rates compared with favorable prognoses.

miRNA	FDR	Log2 FC
Upregulated		
hsa-miR-30c-5p	0.045	2.555
hsa-miR-4532	0.045	2.274
hsa-miR-4792	0.043	2.222
hsa-let-7a-5p	0.043	1.628
hsa-let-7b-5p	0.040	1.588
hsa-miR-199a-5p	0.039	1.578
Downregulated		
hsa-miR-7641	0.035	−1.161
hsa-miR-1307-3p	0.032	−1.229
hsa-miR-199b-5p	0.031	−1.274
hsa-miR-1246	0.024	−1.281
hsa-miR-23b-3p	0.022	−1.369
hsa-miR-145-5p	0.019	−1.610
hsa-miR-4497	0.015	−1.650

FDR: false discovery rate; FC: fold change.

**Table 2:** Top-30 hub genes based on the degree centrality in the PPI network associated with early-stage OSCC patients with dismal prognoses.

Gene ID	Degree	Betweenness
TP53	187	0.094
ACTB	168	0.071
MYC	161	0.055
EGFR	137	0.034
SRC	135	0.037
HRAS	132	0.036
PTEN	132	0.028
CCND1	126	0.029
KRAS	122	0.025
NOTCH1	121	0.020
VEGFA	120	0.018
ESR1	115	0.024
CDH1	115	0.020
IL6	114	0.018
HIF1A	114	0.014
CASP3	109	0.018
ERBB2	109	0.014
SIRT1	96	0.022
SMAD4	92	0.017
SOX2	90	0.009
EZH2	88	0.014
SMAD3	87	0.012
MDM2	84	0.013
CD44	83	0.006
CDKN1A	81	0.011
GSK3B	80	0.011
CDK4	78	0.007
POU5F1	78	0.005
NANOG	77	0.008
STAT1	70	0.009

PPI: protein-protein interaction; OSCC: oral squamous cell carcinoma.

**Table 3:** A total of 15 genes were found to be prognostic markers in patients with OSCC.

Gene symbols	HR (high)	*p* (log-rank test)	*p* (HR)
EIF2S1	1.7	0.00016	0.0002
CAV1	1.5	0.0016	0.0016
SERPINE1	1.5	0.0024	0.0025
ACTB	1.5	0.0054	0.0057
SMAD3	1.4	0.017	0.018
HMGA2	1.4	0.017	0.018
MYC	1.3	0.028	0.029
IL6	1.3	0.029	0.029
HSPA4	1.3	0.035	0.036
HSPA5	1.3	0.042	0.043
PDGFRA	0.74	0.024	0.024
E2F2	0.74	0.024	0.024
ESR1	0.74	0.024	0.025
DDX17	0.74	0.027	0.027
AGO4	0.74	0.028	0.028

OSCC: oral squamous cell carcinoma.

**Table 4:** Structural information of the receptors used for molecular docking analysis.

HGNC	UniProt ID	Protein name	Source of the 3D structure and no. of residues	Strategy (or the source used) for identifying binding site residues	Binding site residues	Grid box settings	References
SERPINE1	P05121	Plasminogen activator inhibitor 1	RCSB (PDB ID, 1A7C; chain, A); no. of residues, 379	Analyzing interactions between SERPINE1 residues and the ligand (pentapeptide) inside the 1A7C file using DSV	Leu160; Arg162; Leu163; Val164; Leu165; Gln301; Phe306; Glu313; Pro314; Leu315; His316; Val317; Ala318; Gln319	*X*-dimension, 60; *Y*-dimension, 60; *Z*-dimension, 60; *X*-center, 23.904; *Y*-center, 23.032; *Z*-center, 0.217	[56, 57]

ACTB	P60709	Actin, cytoplasmic 1	RCSB (PDB ID, 6NBW; chain A); no. of residues, 374	Analyzing interactions between ACTB residues and the ligand (ATP) inside the 6NBW file using DSV	Ser14; Gly15; Met16; Lys18; Gly74; Gly156; Asp157; Gly158; Val159; Gly182; Lys213; Gly301; Gly302; Met305; Tyr306; Lys336	*X*-dimension, 68; *Y*-dimension, 48; *Z*-dimension, 60; *X*-center, -15.029; *Y*-center, -22.916; *Z*-center, 18.971	[56, 58]

SMAD3	P84022	Mothers against decapentaplegic homolog 3	RCSB (PDB ID, 5ODG; chain A); no. of residues, 128	10.1038/s41467-017-02054-6	Lys33; Lys41; Leu71; Asp72; Arg74; Gln76; Ser78; His79; Lys81	*X*-dimension, 66; *Y*-dimension, 60; *Z*-dimension, 60; *X*-center, 2.592; *Y*-center, 28.185; *Z*-center, 3.042	[56, 59]

HMGA2	P52926	High mobility group protein HMGI-C	Modeling using I-TASSER; no. of residues, 109	UniProt database: interacting residues with E4F1	Ser44; Pro45; Lys46; Arg47; Pro48; Arg49; Gly50; Arg51; Pro52; Lys53; Gly54; Ser55; Lys56; Asn57; Lys58; Ser59; Pro60; Ser61; Lys62; Ala63	*X*-dimension, 84; *Y*-dimension, 60; *Z*-dimension, 64; *X*-center, 58.836; *Y*-center, 61.746; *Z*-center, 70.513	[41, 60]

MYC	P01106	Myc proto-oncogene protein	RCSB (PDB ID, 5I4Z; chain B); no. of residues, 118	10.1186/s12906-022-03662-6	Ile34; Pro35; Glu36; Lys51; Ala54; Tyr55; Ser58	*X*-dimension, 50; *Y*-dimension, 36; *Z*-dimension, 42; *X*-center, 35.114; *Y*-center, 24.691; *Z*-center, 6.372	[56, 61, 62]

EIF2S1	P05198	Eukaryotic translation initiation factor 2 subunit 1	RCSB (PDB ID, 1KL9; chain A); no. of residues, 182	CASTp server	Tyr8; Gln9; His10; Phe12; Pro13; Glu14; Asp17; Val18; Val19; Glu37; Tyr38; Lys100; Trp135; Asp138; Asp139; Lys142; Pro144	*X*-dimension, 62; *Y*-dimension, 62; *Z*-dimension, 52; *X*-center, 7.405; *Y*-center, 1.699; *Z*-center, 7.342	[56, 63, 64]

HSPA4	P34932	Heat shock 70 kDa protein 4	Modeling using SWISS-MODEL; template, 2QXL.1.A; identity, 39.45%; no. of residues, 840	CASTp server	Arg69; His71; Gly72; Arg73; Ala74; Asp77; Pro78; Phe79; Ala82; Glu83; Leu94; Pro95; Thr96; Leu98; Thr113; Glu115; Gln116; Cys146; Phe147; Tyr148; Thr149; Asp150; Ala151; Glu152; Arg153; Ser155; Asp158; Ala159; Ile162; Arg169; Leu170; Met171; Asp172; Thr174; Thr175; Ser211; Lys221; Val222; Leu223; Thr225; Ala226; Phe227; Thr229; Thr230; Ser323; Val324; Glu326; Gln327; Thr328; Lys329; Ile394; Thr395; Asp396; Val397; Val398; Pro399; Tyr400; Pro401; Ile402; Phe420; Lys422; Asn423; His424; Ala425; Ala426; Pro427; Tyr446; Ser448; Gln450; Asp451; Leu452; Pro453; Tyr454; Pro455; Asp456; Ala458; Ile459; Val484; Val486; His487; Gly488; Phe490; Trp587; Ile589; Asp590; Met593; Leu594; Tyr597	*X*-dimension, 104; *Y*-dimension, 86; *Z*-dimension, 126; *X*-center, 73.243; *Y*-center, 102.156; *Z*-center, -20.235	[64, 65]

HSPA5	P11021	Endoplasmic reticulum chaperone BiP	RCSB (PDB ID, 6DO2; chain A); no. of residues, 382	Analyzing interactions between HSPA5 residues and the ligand (PDB ID, H5V) inside the 6DO2 file using DSV	Tyr39; Lys296; Arg297; Ser300; Gly364; Arg367; Asp391	*X*-dimension, 50; *Y*-dimension, 44; *Z*-dimension, 46; *X*-center, -11.523; *Y*-center, -7.916; *Z*-center, 3.79	[56]

IL6	P05231	Interleukin-6	RCSB (PDB ID, 1ALU; chain A); no. of residues, 186	CASTp server	Glu95; Val96; Leu98; Glu99; Gln116; Lys120; Pro141; Asn144	*X*-dimension, 32; *Y*-dimension, 50; *Z*-dimension, 54; *X*-center, 10.018; *Y*-center, -20.018; *Z*-center, 17.868	[56, 64, 66]

DSV: discovery studio visualizer.

**Table 5:** AutoDock 4.0 employed the incorporation of Kollman charges onto the receptors.

Protein	Kollman charges
SERPINE1	9.771
ACTB	−5.742
SMAD	14.054
HMGA2	13
MYC	6.26
EIF2S1	−7.831
HSPA4	−14
HSPA5	2
IL6	1.761

**Table 6:** The Gibbs free energy of binding between SBG components and nine markers associated with poor prognosis in patients with OSCC was calculated in kcal/mol using the AutoDock 4.0 tool.

		Baicalein	Wogonin	Oroxylin-A	Salvigenin	Norwogonin
Targets	SERPINE1	−9.56	−10.02	−9.14	−9.61	−9.8
	ACTB	−9.84	−9.22	−9.25	−8.77	−7.5
	SMAD	−6.51	−6.83	−6.13	−6.1	−6.88
	HMGA2	−9.26	−8.8	−9.08	−8.63	−9.48
	MYC	−6.7	−7.13	−6.35	−5.34	−0.62
	EIF2S1	−9.02	−8.91	−7.7	−6.55	−8.93
	HSPA4	−8.85	−9.09	−8.75	−8.87	−5.88
	HSPA5	−8.44	−7.97	−8.43	−8.14	−8.93
	IL6	−7.72	−7.68	−7.04	−6.83	−8.06

SBG: *Scutellaria baicalensis* Georgi; OSCC: oral squamous cell carcinoma.

**Table 7:** The *K*_*i*_ values between SBG components and nine markers associated with poor prognosis in patients with OSCC were calculated using the AutoDock 4.0 tool.

		Baicalein	Wogonin	Oroxylin-A	Salvigenin	Norwogonin
Targets	SERPINE1	98.59 nM	45.08 nM	200.40 nM	90.23 nM	65.63 nM
	ACTB	60.94 nM	173.06 nM	166.22 nM	373.79 nM	3.18 *μ*M
	SMAD	16.95 *μ*M	9.80 *μ*M	31.98 *μ*M	33.88 *μ*M	9.04 *μ*M
	HMGA2	164.13 nM	356.87 nM	220.47 nM	473.27 nM	112.03 nM
	MYC	12.32 *μ*M	5.97 *μ*M	22.15 *μ*M	121.01 *μ*M	348.41 mM
	EIF2S1	244.07 nM	296.78 nM	2.28 *μ*M	15.89 *μ*M	286.11 nM
	HSPA4	324.93 nM	218.93 nM	385.93 nM	315.78 nM	49.05 *μ*M
	HSPA5	650.41 nM	1.43 *μ*M	662.12 nM	1.07 *μ*M	286.07 nM
	IL6	2.21 *μ*M	2.37 *μ*M	6.89 *μ*M	9.93 *μ*M	1.23 *μ*M

SBG: *Scutellaria baicalensis* Georgi; OSCC: oral squamous cell carcinoma; *K*_*i*_: inhibition constant.

## Data Availability

The datasets used and/or analyzed during the current study are available from the corresponding author upon reasonable request.

## Supplementary Materials

Supplementary 1 Supplementary Table 1: a total of 88 hub genes in the PPI network associated with early-stage OSCC patients with dismal prognoses.

Supplementary 2 The detailed overview of all model assessment analyses.
