# Supplementary material for: Mechanisms Involved in Therapeutic Effects of Scutellaria baicalensis Georgi in Oral Squamous Cell Carcinoma Based on Systems Biology and Structural Bioinformatics Approaches
Source: Biomed Res Int. 2024 Jan 30;2024:1236910. doi: 10.1155/2024/1236910 (PMC10846925; doi:10.1155/2024/1236910)
Supplement: Supplementary 1 — Supplementary Table 1: a total of 88 hub genes in the PPI network associated with early-stage OSCC patients with dismal prognoses. [file 1236910.f1.docx]

**Supplementary Table 1**. A total of 88 hub genes in the PPI network associated with early-stage OSCC patients with dismal prognoses.

| **Gene ID** | **Degree** | **Betweenness** |
| --- | --- | --- |
| TP53 | 187 | 0.094 |
| ACTB | 168 | 0.071 |
| MYC | 161 | 0.055 |
| EGFR | 137 | 0.034 |
| SRC | 135 | 0.037 |
| HRAS | 132 | 0.036 |
| PTEN | 132 | 0.028 |
| CCND1 | 126 | 0.029 |
| KRAS | 122 | 0.025 |
| NOTCH1 | 121 | 0.020 |
| VEGFA | 120 | 0.018 |
| ESR1 | 115 | 0.024 |
| CDH1 | 115 | 0.020 |
| IL6 | 114 | 0.018 |
| HIF1A | 114 | 0.014 |
| CASP3 | 109 | 0.018 |
| ERBB2 | 109 | 0.014 |
| SIRT1 | 96 | 0.022 |
| SMAD4 | 92 | 0.017 |
| SOX2 | 90 | 0.009 |
| EZH2 | 88 | 0.014 |
| SMAD3 | 87 | 0.012 |
| MDM2 | 84 | 0.013 |
| CD44 | 83 | 0.006 |
| CDKN1A | 81 | 0.011 |
| GSK3B | 80 | 0.011 |
| CDK4 | 78 | 0.007 |
| POU5F1 | 78 | 0.005 |
| NANOG | 77 | 0.008 |
| STAT1 | 70 | 0.009 |
| SNAI2 | 70 | 0.005 |
| HSPA4 | 69 | 0.023 |
| SMARCA4 | 69 | 0.016 |
| KIT | 69 | 0.005 |
| DNMT1 | 66 | 0.011 |
| CDH2 | 66 | 0.005 |
| HDAC2 | 65 | 0.008 |
| CAV1 | 65 | 0.004 |
| PTGS2 | 63 | 0.011 |
| CCNA2 | 63 | 0.007 |
| CASP8 | 63 | 0.005 |
| DICER1 | 62 | 0.014 |
| NRAS | 61 | 0.007 |
| IRS1 | 60 | 0.014 |
| RUNX2 | 60 | 0.006 |
| YAP1 | 59 | 0.009 |
| CASP9 | 59 | 0.004 |
| RPS6KB1 | 57 | 0.004 |
| SP1 | 55 | 0.008 |
| RB1 | 55 | 0.006 |
| ACTG1 | 54 | 0.008 |
| CCND2 | 54 | 0.007 |
| HSPA5 | 53 | 0.024 |
| APP | 52 | 0.014 |
| AURKB | 50 | 0.018 |
| ITGB3 | 49 | 0.009 |
| AURKA | 49 | 0.009 |
| E2F1 | 49 | 0.007 |
| HMGA2 | 49 | 0.006 |
| NFKB1 | 48 | 0.015 |
| NOTCH2 | 48 | 0.006 |
| BECN1 | 48 | 0.006 |
| WNT2 | 47 | 0.012 |
| VIM | 46 | 0.007 |
| SERPINE1 | 46 | 0.006 |
| PDGFRA | 46 | 0.005 |
| IKBKB | 46 | 0.004 |
| UBE2I | 42 | 0.009 |
| EIF4E | 41 | 0.009 |
| MUC1 | 40 | 0.014 |
| SOCS3 | 40 | 0.006 |
| CDC25A | 39 | 0.005 |
| VDR | 38 | 0.006 |
| AGO4 | 37 | 0.005 |
| AGO1 | 37 | 0.004 |
| CBFB | 36 | 0.006 |
| PIK3R2 | 35 | 0.005 |
| TYMS | 33 | 0.011 |
| E2F2 | 33 | 0.004 |
| SMARCA2 | 32 | 0.011 |
| NFATC1 | 30 | 0.004 |
| PRKACB | 28 | 0.010 |
| IDH1 | 28 | 0.010 |
| CFTR | 28 | 0.009 |
| DDX17 | 26 | 0.016 |
| EIF2S1 | 26 | 0.011 |
| RRM2 | 26 | 0.004 |
| VHL | 24 | 0.005 |

PPI, protein-protein interaction; OSCC, oral squamous cell carcinoma.
