# Supplementary material for: Mechanisms Involved in Therapeutic Effects of Scutellaria baicalensis Georgi in Oral Squamous Cell Carcinoma Based on Systems Biology and Structural Bioinformatics Approaches
Source: Biomed Res Int. 2024 Jan 30;2024:1236910. doi: 10.1155/2024/1236910 (PMC10846925; doi:10.1155/2024/1236910)
Supplement: Supplementary 2 — The detailed overview of all model assessment analyses. [file 1236910.f2.docx]

**Model assessment analysis for HMGA2, HSPA4, and CAV1**

**HMGA2**

**
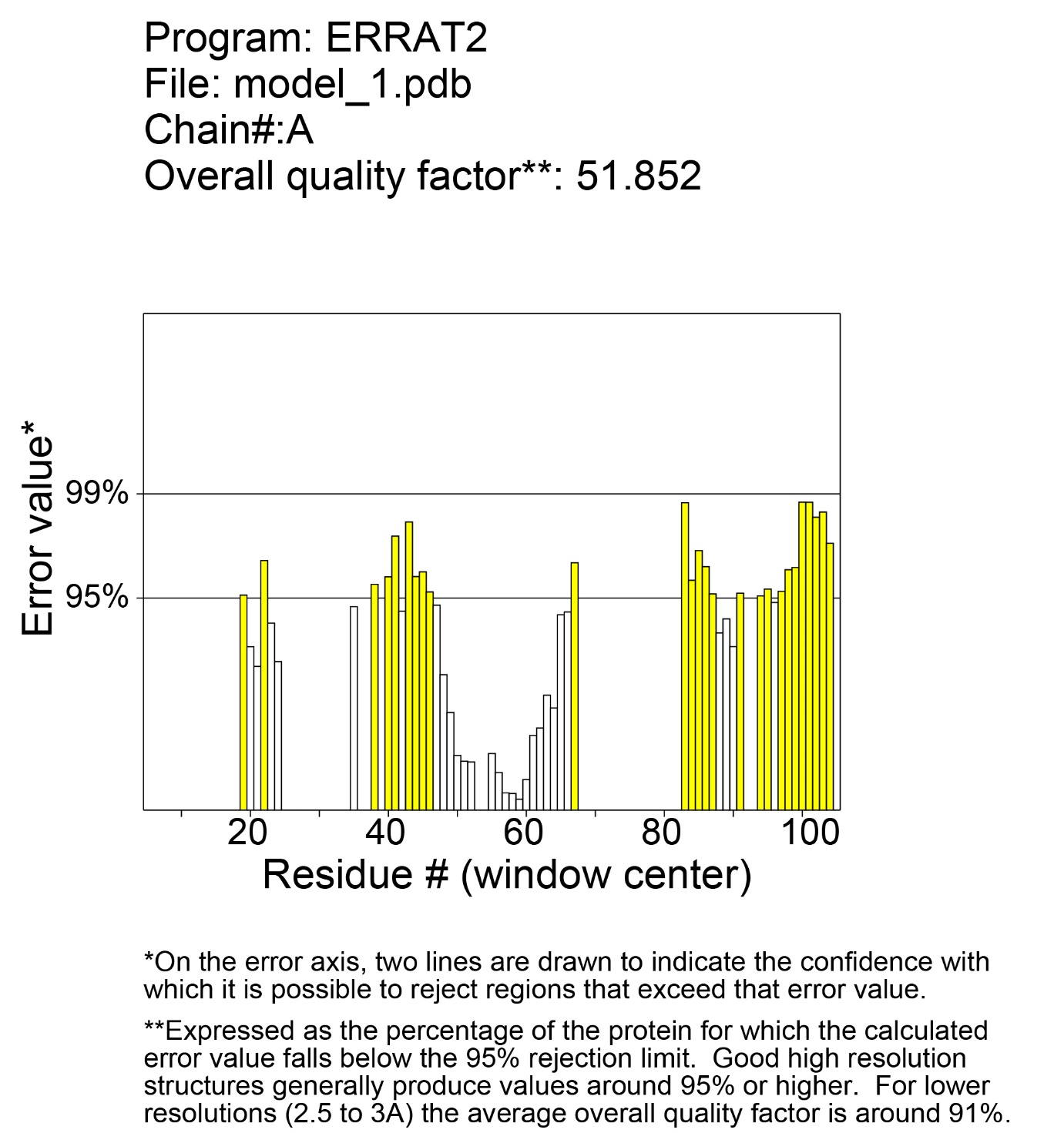
**

**
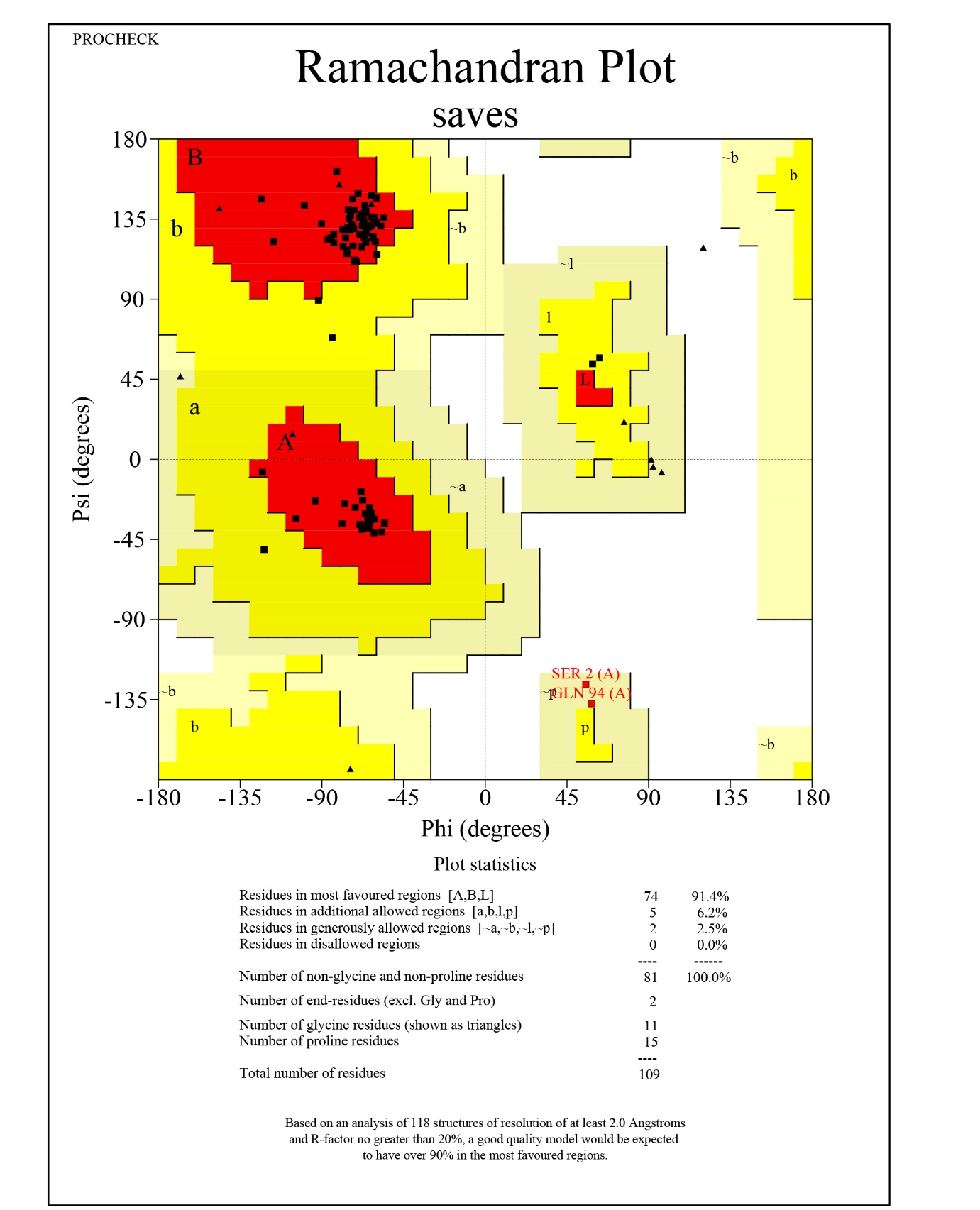
**

**VERIFY3D**

84.40% of the residues have
averaged 3D-1D score >= 0.1

**Pass**

At least 80% of the amino acids have scored >= 0.1 in the 3D/1D profile

**
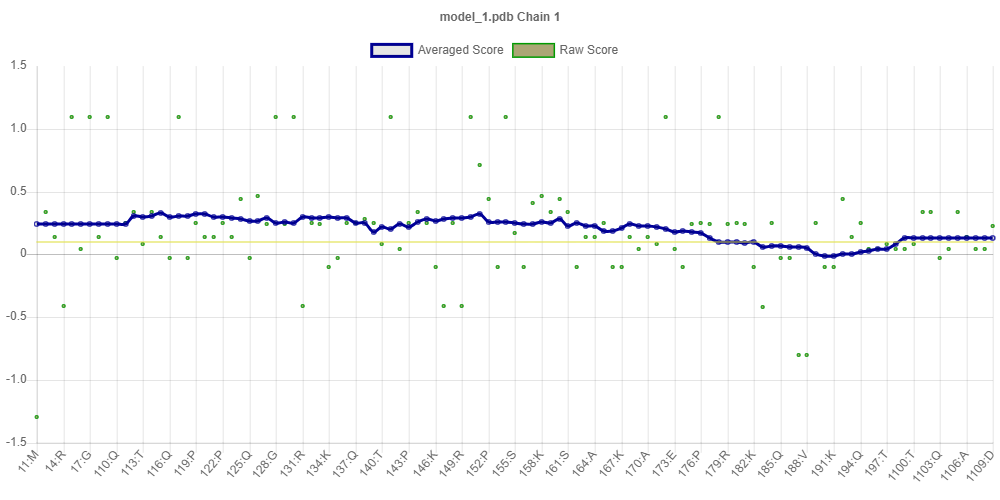
**

**
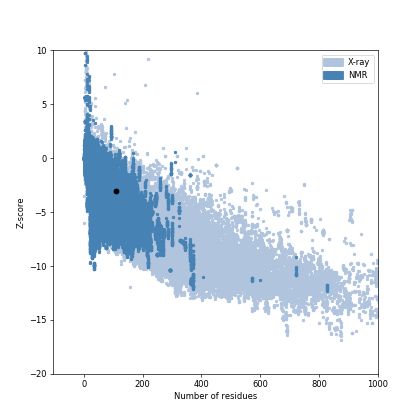
**

**HSPA4**


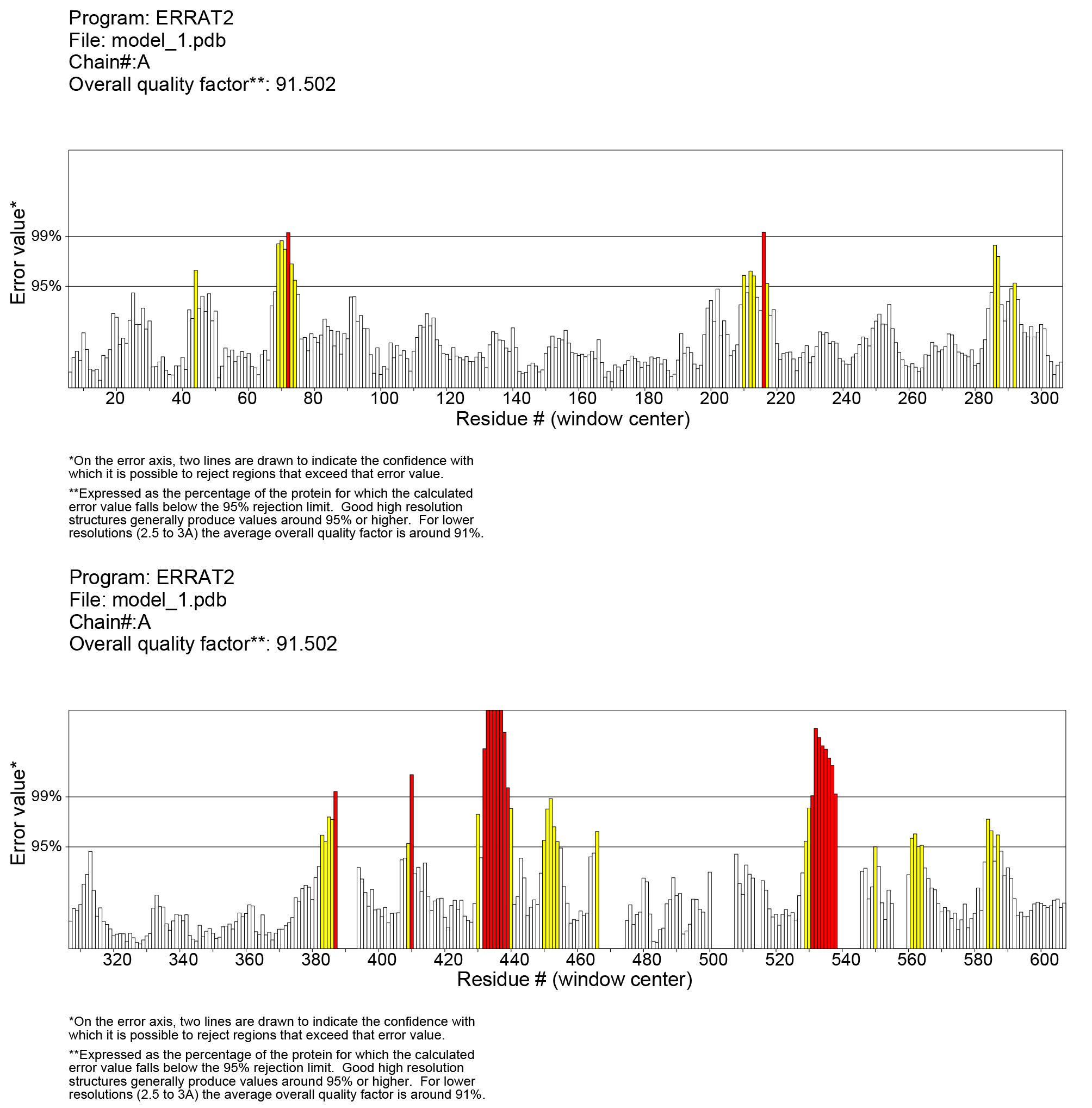


**
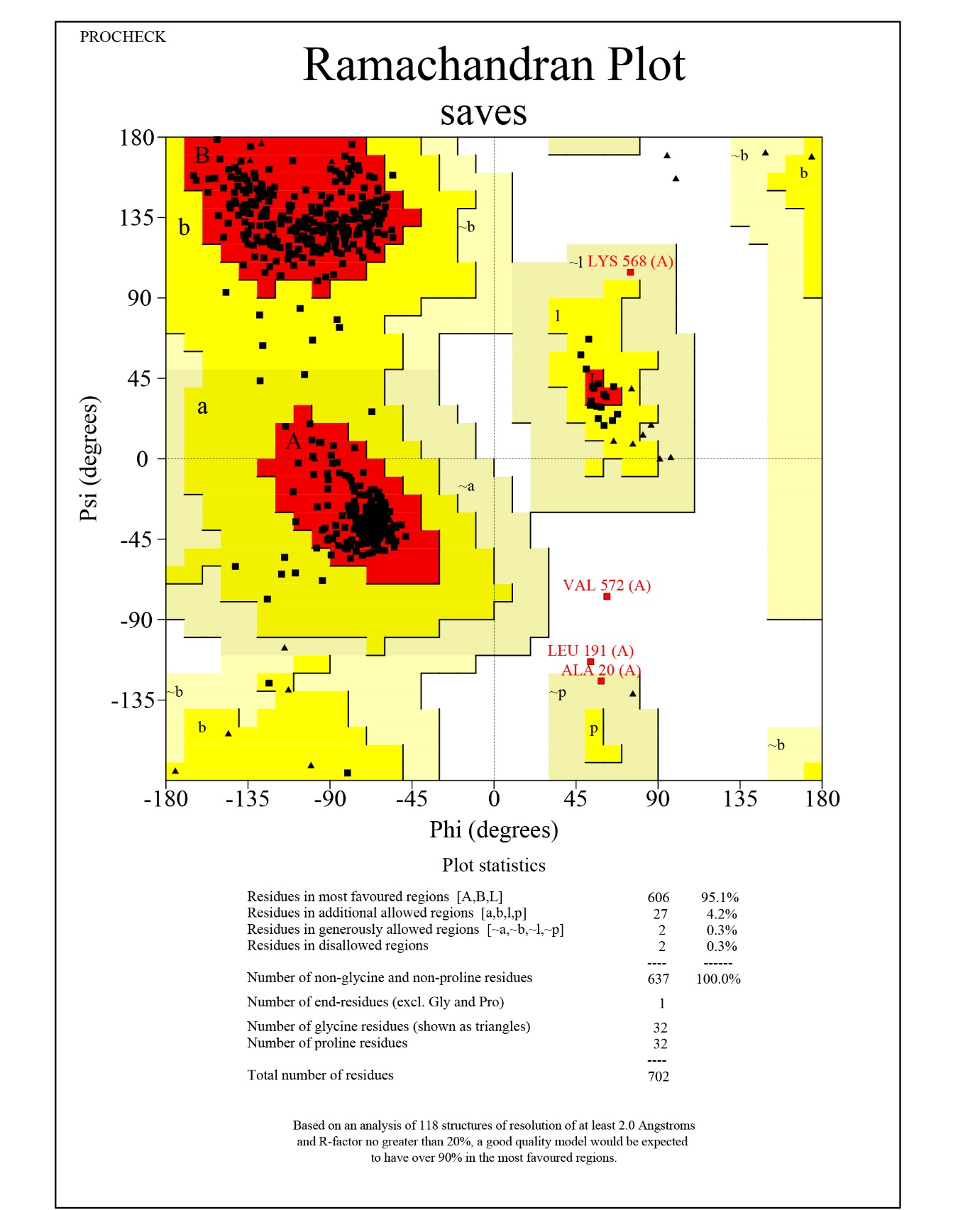
**

**VERIFY3D**

76.50% of the residues have
averaged 3D-1D score >= 0.1

**Fail**

Fewer than 80% of the amino acids have scored >= 0.1 in the 3D/1D profile.

**
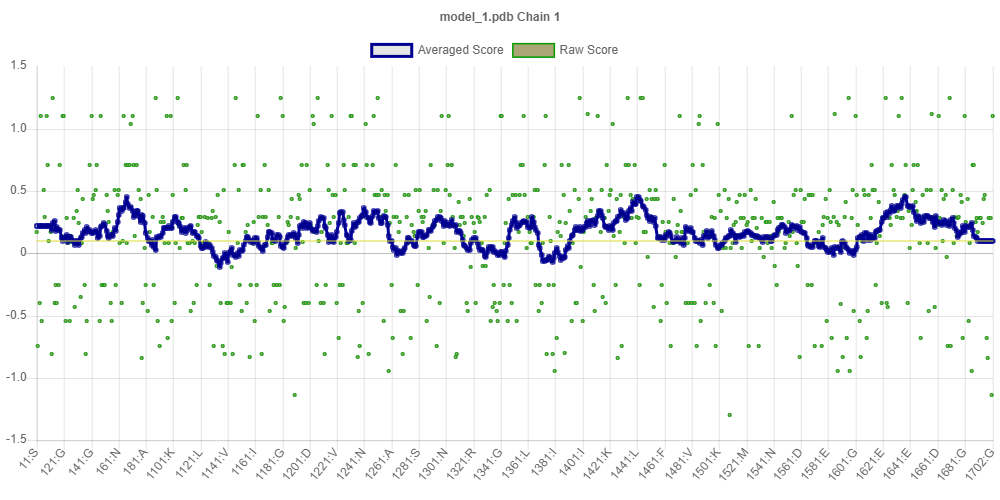
**

**
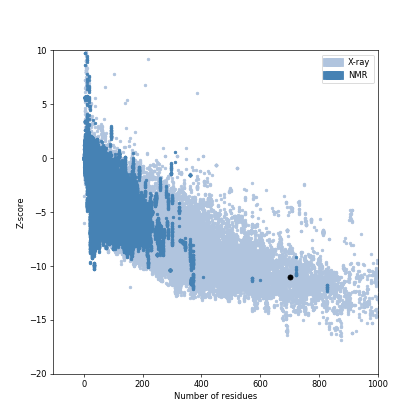
**

**CAV1**

**
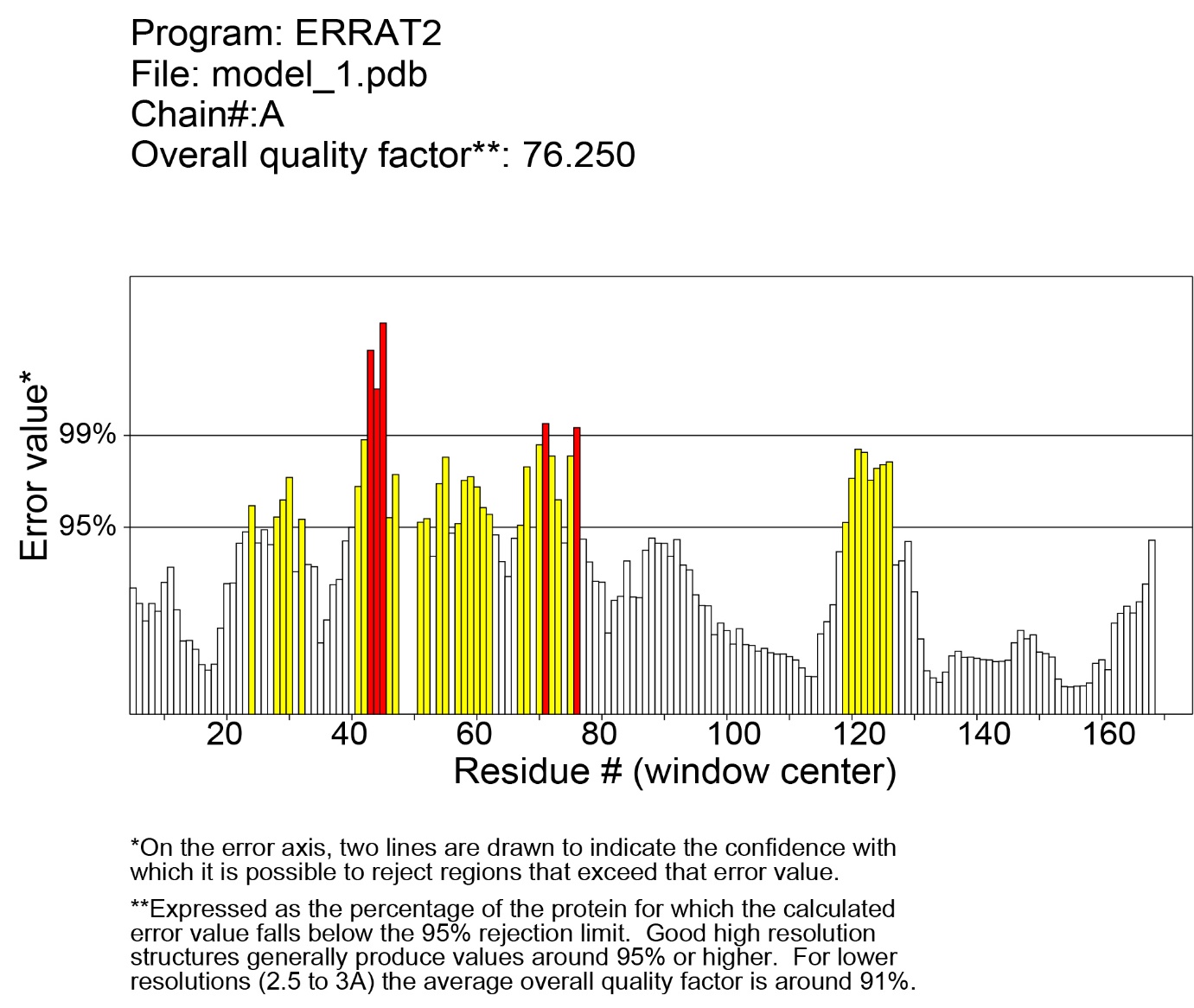
**

**
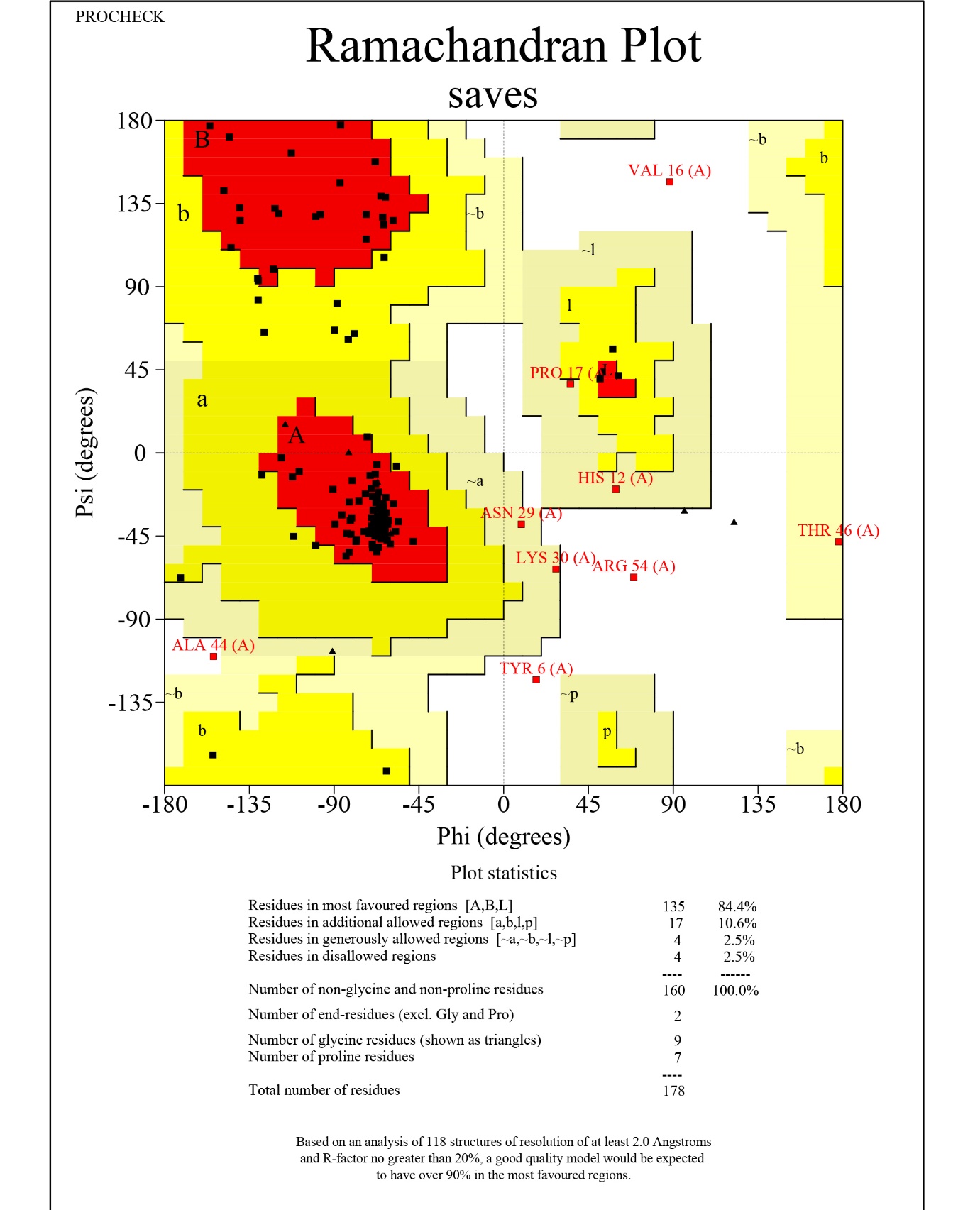
**

**VERIFY3D**

14.04% of the residues have
averaged 3D-1D score >= 0.1

**Fail**

Fewer than 80% of the amino acids have scored >= 0.1 in the 3D/1D profile.

**
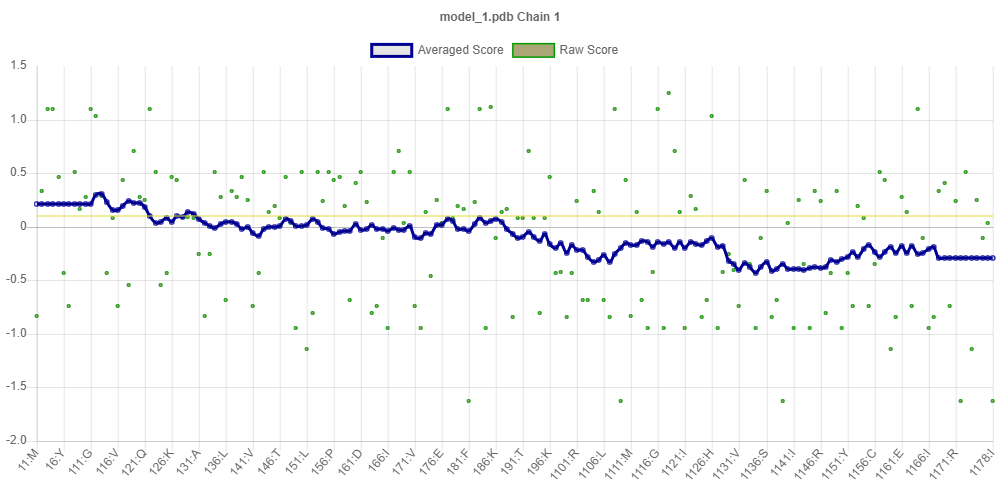
**

**
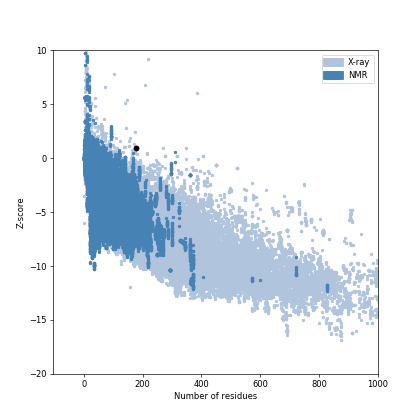
**
